# The translation inhibitor silvestrol exhibits direct anti-tumor activity while preserving innate and adaptive immunity against EBV-driven lymphoproliferative disease

**DOI:** 10.18632/oncotarget.2098

**Published:** 2014-06-12

**Authors:** John T. Patton, Mark E. Lustberg, Gerard Lozanski, Sabrina L. Garman, William H. Towns, Callie M. Drohan, Amy Lehman, Xiaoli Zhang, Brad Bolon, Li Pan, A. Douglas Kinghorn, Michael R. Grever, David M. Lucas, Robert A. Baiocchi

**Affiliations:** ^1^ Division of Hematology, Department of Internal Medicine, College of Medicine, The Ohio State University, Columbus, Ohio, USA; ^2^ Division of Infectious Disease, The Ohio State University, Columbus, Ohio, USA; ^3^ Department of Pathology, The Ohio State University, Columbus, Ohio, USA; ^4^ Center for Biostatistics, The Ohio State University, Columbus, Ohio, USA; ^5^ Comparative Pathology and Mouse Phenotyping Shared Resource, Comprehensive Cancer Center, The Ohio State University, Columbus, Ohio, USA; ^6^ Division of Medicinal Chemistry and Pharmacognosy, College of Pharmacy, The Ohio State University, Columbus, Ohio, USA

**Keywords:** Lymphoproliferative disease, EBV, silvestrol, immunomodulation

## Abstract

Treatment options for patients with Epstein-Barr Virus-driven lymphoproliferative diseases (EBV-LPD) are limited. Chemo-immunotherapeutic approaches often lead to immune suppression, risk of lethal infection and EBV reactivation, thus it is essential to identify agents that can deliver direct anti-tumor activity while preserving innate and adaptive host immune surveillance. Silvestrol possesses direct anti-tumor activity in multiple hematologic malignancies while causing minimal toxicity to normal mononuclear cells. However, the effects of silvestrol on immune function have not been described. We utilized in vitro and in vivo models of EBV-LPD to simultaneously examine the impact of silvestrol on both tumor and normal immune function. We show that silvestrol induces direct anti-tumor activity against EBV-transformed lymphoblastoid cell lines (LCL), with growth inhibition, decreased expression of the EBV oncogene latent membrane protein-1, and inhibition of the downstream AKT, STAT1 and STAT3 signaling pathways. Silvestrol promoted potent indirect anti-tumor effects by preserving expansion of innate and EBV antigen-specific adaptive immune effector subsets capable of effective clearance of LCL tumor targets in autologous co-cultures. In an animal model of spontaneous EBV-LPD, silvestrol demonstrated significant therapeutic activity dependent on the presence of CD8-positive T-cells. These findings establish a novel immune-sparing activity of silvestrol, justifying further exploration in patients with EBV-positive malignancies.

## INTRODUCTION

Epstein-Barr Virus (EBV) is an oncogenic B-lymphotropic virus associated with Burkitt's lymphoma, non-Hodgkin's and Hodgkin's lymphomas, nasopharyngeal and gastric carcinomas, and post-transplant lymphoproliferative disease (LPD) [1]. Following primary infection, the virus establishes persistent, life-long latency in the B-cell compartment of the human host. This virus/host coexistence is controlled by a highly efficient antigen-specific adaptive immune response that protects immune-competent individuals from EBV-driven pathology. EBV-seropositive individuals who become immunocompromised are at risk for EBV reactivation and development of aggressive B-cell lymphomas. Current treatments for patients with EBV-driven lymphomas are of limited benefit and lead to further immune suppression, risk of opportunistic infections, and a loss of EBV-specific immunity due to dysregulation of immune surveillance [2]. Therefore, novel treatment approaches that target EBV-driven cancers while maintaining normal immune function are in great demand.

Silvestrol [3] is a unique agent that possesses anti-tumor activity in multiple cancer models [4-8]. This capability is attributed to inhibition of translation initiation, which occurs when silvestrol induces aberrant dimerization of the RNA helicase eIF4A with capped mRNA. This effect interferes with normal recruitment of mRNA to the eIF4F initiation complex, thus preventing the rapid synthesis of pro-survival and pro-growth proteins and leading to tumor cell death via caspase-dependent apoptosis [9-12]. Our group reported that silvestrol shows *in vivo* activity in the B-cell malignancies chronic lymphocytic leukemia, acute lymphoblastic lymphoma [13] and mantle cell lymphoma [14], and also that silvestrol appears to be selectively cytotoxic to malignant B-cells while sparing normal lymphocytes [13]. To date, however, the effects of silvestrol on normal immune function have not been evaluated.

Here we show that silvestrol promotes direct anti-tumor activity against EBV-LPD by blocking oncogenic pathways driven by the EBV gene product, latent membrane protein-1 (LMP-1). Furthermore, we demonstrate that silvestrol preserves the anti-tumor function of innate immune effectors as well as antigen-specific adaptive immune effectors in both *in vitro* and *in vivo* models of EBV-LPD. This highly unusual activity suggests that silvestrol may provide an entirely new immune-potentiating therapeutic strategy for this histologic subset of aggressive lymphomas.

## RESULTS

### Silvestrol promotes direct anti-tumor activity against LCL

We first evaluated silvestrol's direct anti-tumor activity in LCL derived from malignant EBV-LPD tumors that spontaneously developed in SCID mice engrafted with PBMC from EBV-seropositive donors [15, 16, 21]. Six different LCL were plated in the presence or absence of silvestrol, and cell viability (annexin/PI negativity; Supplementary Figure 1A) and growth inhibition (MTS assay; Supplementary Figure 1B) were evaluated at 24, 72, and 120 hr. Moderate but significant anti-tumor activity was noted both in growth inhibition and viability assays (p<0.001 and p=0.006, respectively, in silvestrol treated vs. vehicle control), with a 50% growth inhibitory concentration (IC_50_) of approximately 40 nM at 72 hr. Recent pharmacokinetic work in mice indicates that a 10 nM plasma concentration of silvestrol is attainable *in vivo* [22]. Therefore, 10 nM and lower doses were used in subsequent studies.

### Silvestrol induces LMP-1 depletion in LCL

**Figure 1:**
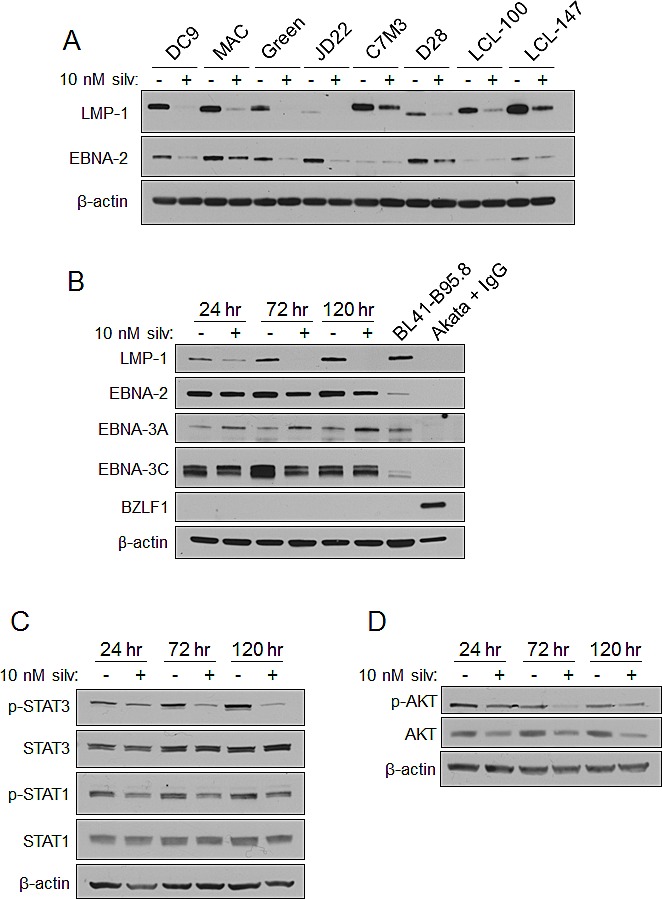
Silvestrol modulates EBV LMP-1 and LMP-1-driven signaling pathways in LCL (A) LCL (N=8) were incubated with 0 or 10 nM silvestrol for 72 hr, and whole cell lysates were immunoblotted for LMP-1 and EBNA-2. β-actin was included as a loading control. (B) Lysates from LCL incubated 24, 72, and 120 hr with 0 or 10 nM silvestrol were evaluated by immunoblot. Akata cells (latency I) were treated with 7.5μg/ml anti-IgG for 24 hr to induce lytic cycle. BL41-B95.8 were incubated for 24 hr untreated. Results shown are representative of 3 different LCL. (C) LCL incubated as in (B) were immunoblotted for phosphorylated and total STAT1 and STAT3. Results were representative of 3 LCL. (D) LCL incubated as in (B) were immunoblotted for phosphorylated and total AKT. Results were representative of 3 LCL.

The virally-encoded transmembrane oncoprotein LMP-1 acts as a constitutively active receptor of the TNF-R family [23], promotes multiple growth and survival pathways, suppresses immune-activating cytokines, and is essential for B-cell transformation [24, 25]. These properties make it a potentially valuable therapeutic target for LMP-1-expressing Type II or III EBV-driven malignancies [26-30]. Therefore, we evaluated expression of LMP-1 protein, as well as its trans-activator EBNA-2, in eight LCL lines (including the six lines used in the viability and proliferation assays above) by immunoblot 72 hr after treating with silvestrol (Figure 1A). We observed a notable drop in LMP-1 expression across all LCL tested, and a corresponding decrease in EBNA-2 in six of the eight. As shown in a representative LCL (DC9; Figure 1B), LMP-1 levels fall incrementally as a function of time after a single 10 nM dose of silvestrol, even though the effect on EBNA-2 in this LCL was minor. Silvestrol had varying effects on the latent EBV gene products EBNA-3A and -3C, however, and did not induce the expression of the lytic transcription factor BZLF-1 (Figure 1B). Lysates from Akata cells (Type I latency) incubated with anti-IgG to induce lytic cycle and BL41-B95.8 cells (Type III latency) were included as controls [31-33].

LMP-1 is known to constitutively activate multiple pro-survival signaling pathways including NF-κB, PI3K/AKT, STAT1 and STAT3 through its cytoplasmic C-terminal-activating regions (CTAR1 and 2), biologically mimicking the TNF family receptor CD40 [25, 34-37]. Thus, LMP-1 promotes tumor cell survival and growth through diverse mechanisms. To investigate the effects of silvestrol on these LMP-1-induced pathways, LCL were incubated for 24, 72 and 120 hr with vehicle or 10 nM silvestrol and cell extracts were analyzed by immunoblot. While total STAT1 and STAT3 levels remained unchanged, the levels of their phosphorylated (activated) forms decreased (Figure 1C). Decreases of both total AKT and its activated, phosphorylated form (Figure 1D) were also observed. Unexpectedly, NF-κB p65 phosphorylation increased with silvestrol treatment, suggesting activation, although total p65 levels remained relatively unchanged (Supplementary Figure 2A). Total levels of NF-κB components p50, p105 and IκBα were unchanged, as were NF-kB targets Bcl-2 and Bax, and despite the silvestrol-induced phosphorylation of p65, none of this phosphorylated p65 was evident in the nuclear fraction (Supplementary Figure 2A, 2B, 2C). Together, these data suggest that changes in STAT and AKT, rather than NF-kB, may underlie the direct anti-tumor effects of silvestrol.

We next examined the effects of silvestrol on known short half-life proteins, as we and others have reported in other cell types [12, 14]. Cyclin D3 and CDK4 were each notably reduced by 24 hr in three out of four LCL tested (Supplementary Figure 2D). It is unclear why this effect differed in one the four LCL (JD22), although this relative lack of protein effect also corresponded with reduced growth inhibition by silvestrol in this LCL (MTS assay; not shown). As an expected consequence of the silvestrol-mediated depletion of cyclin D3 and CDK4, phosphorylation of Rb was notably diminished (Supplementary Figure 2E), consistent with our earlier findings [14].

### Immune effector function is preserved in the presence of silvestrol in irradiated co-cultures

To explore the immune modulatory activity of silvestrol in EBV-LPD, co-cultures (CoCx) were created by lethally irradiating LCL and plating in the presence of autologous PBMC (1:1 ratio). Under these conditions, memory adaptive components of PBMC become activated and expand in response to the antigenic stimuli from the LCL [15, 16, 21]. CoCx created using three LCL and their respective autologous PBMC were treated once with 0 (vehicle control), 2, 5, or 10 nM silvestrol and incubated for 14 days. Although the total cell numbers expanded in CoCx in the presence of 5 or 10 nM silvestrol appeared to be lower than in the vehicle-treated CoCx, these differences did not reach significance, and all CoCx conditions exhibited total cell numbers equal to or greater than unstimulated PBMCs alone (Supplementary Figure 3A). As no irradiated LCL remain by day 14 (not shown), this result demonstrates that addition of silvestrol still allows for expansion of normal effector populations following exposure to LCL. Immunophenotyping of the resulting populations showed no statistically significant differences in both CD8^+^ cytotoxic T-cells (CTL) and CD4^+^ helper T-cells with silvestrol treatment (Supplementary Figure 3B, 3C; results shown relative to untreated CoCx). Similarly, no significant differences were seen with silvestrol treatment in the LCL-induced expansion of CD56^+^ NK cells, the majority of the expanded population (Supplementary Figure 3D). These results indicate that silvestrol exposure under these conditions is permissive for viability and growth of innate and adaptive immune effector cells.

### Silvestrol leads to depletion of LCL in non-irradiated co-cultures

**Figure 2:**
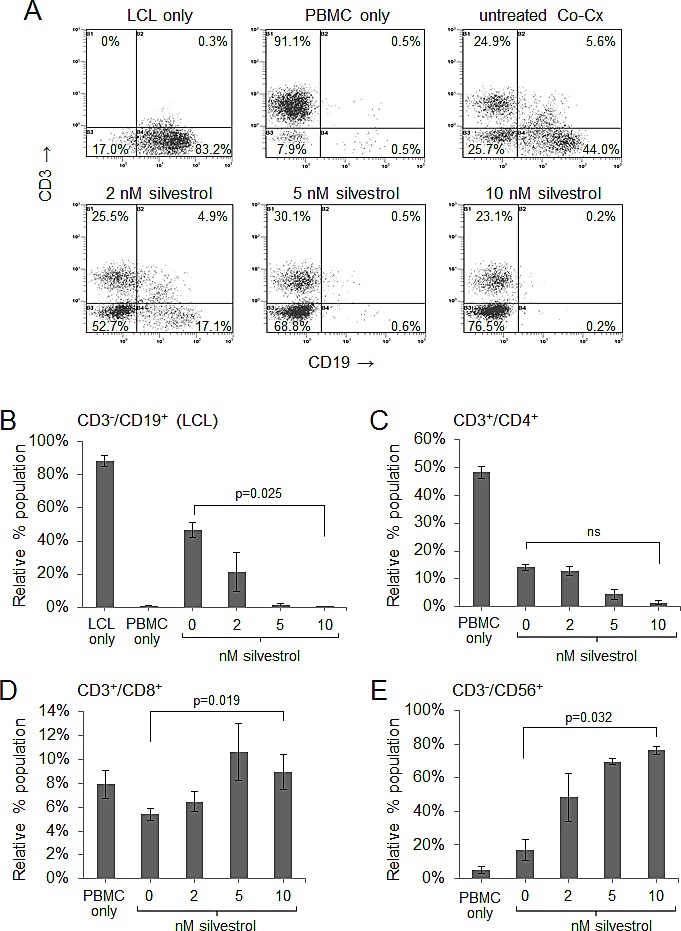

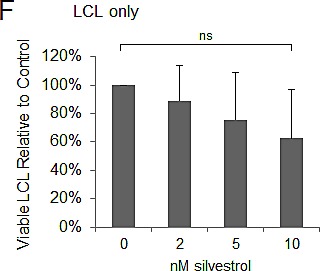
Silvestrol leads to depletion of non-irradiated LCL in co-cultures while permitting expansion of T and NK cells CoCx (N=3) were created by mixing non-irradiated LCL with equal numbers of autologous peripheral blood mononuclear cells (PBMC). CoCx or PBMC alone were incubated in the presence of 10 U/ml IL-2 and given a single dose of 0 (vehicle only), 2, 5, or 10 nM silvestrol. Flow cytometric analysis was conducted on day 10. For all results, live events were gathered by gating on cells negative for the LIVE/DEAD stain. (A) Representative flow cytometry dot plots of mononuclear cells from CoCx. Cells were stained for CD3 (y-axis) and CD19 (x-axis) and gated on live events. LCL (CD3^−^/CD19^+^) are shown in the bottom right quadrant of each panel. (B-E) Data are expressed as percentage of total viable population expressing: (B) CD3^−^/CD19^+^ (LCL); (C) CD3^+^/CD4^+^ (helper T-cells); (D) CD3^+^/CD8^+^ (cytotoxic T cells); (E) CD3^−^/CD56^+^ (NK cells). All results are averages of three individual CoCx; ns = not significant. (F) Four different LCL were cultured using the same conditions as in the above co-cultures, but without the addition of PBMCs, and incubated with a single dose of silvestrol. Viable LCL were enumerated by flow cytometry, using cell counting beads and gating on cells negative for the LIVE/DEAD stain, and are shown relative to the untreated (vehicle) control. Differences with silvestrol treatment (0 versus 10 nM) were not significant (ns).

We next examined how silvestrol impacts viable, non-irradiated LCL in the presence of immune effectors. LCL were incubated 1:1 with their respective autologous PBMC for 10 days after adding a single dose of silvestrol (0, 2, 5 or 10 nM). Figure 2A shows representative flow cytometry data for LCL+PBMC CoCx generated from one donor (data from additional donors are presented in Supplementary Figure 4). LCL cells (CD3^−^/CD19^+^) appear in the bottom right quadrant of each plot; PBMC-derived effector populations (CD19-) appear in the left quadrants. In untreated (vehicle control) conditions, transformed LCL cells proliferated and matched the expansion of effector cell subsets, approximately maintaining the 1:1 ratio (Figure 2A upper right panel; all events gated on the viable population). However, with a one-time addition of silvestrol, a dose-dependent ablation of viable LCL was observed (Figure 2A, lower three panels). Results from three separate experiments are quantified in Figure 2B and demonstrate a significant loss of LCL in the presence of silvestrol (p=0.025 for 0 versus 10 nM). Percentages of CD4+ T-cells appeared to be reduced with silvestrol treatment as well (Figure 2C), although the differences were not statistically significant (p=0.537 for 0 versus 10 nM). In contrast, CD8^+^ T-cells (Figure 2D) and CD56^+^ NK cells (Figure 2E) expanded significantly in the presence of silvestrol (p=0.019 and p=0.032, respectively, for 0 versus 10 nM silvestrol). Importantly, this significant effect was not observed when LCL were incubated without PBMC in 5 or 10 nM silvestrol under otherwise identical conditions (Figure 2F). Although a trend toward lower cell numbers (enumerated using counting beads) was observed in the presence of silvestrol, this effect did not reach significance (p=0.072 for 0 versus 10 nM), consistent with the modest growth inhibitory effects previously mentioned (Supplementary Figure 1).

Similar cultures were set up and treated with a single dose of fludarabine (active metabolite 2-fluoro-ara-A), as this agent has been used clinically to treat LPD. However, unlike silvestrol, 2-fluoro-ara-A treatment resulted in outgrowth of LCL (Supplementary Figure 5A, 5B). It also exhibited indiscriminate cytotoxicity toward the effector populations, leading to dramatically fewer effectors in the 2-fluoro-ara-A treated versus untreated conditions (Supplementary Figure 5B). This is in stark contrast to the nearly 100% viability of effectors in the silvestrol treated CoCx.

We next worked to identify which effector subsets mediate the anti-LCL effect in the presence of silvestrol. Cells expressing CD8 (CTL), CD14 (monocytes) or CD56 (NK cells) were depleted from PBMC using immunomagnetic beads; mock depletion with biotin-only beads was included as a control. Effector cell numbers equivalent to the total number in the mock-depleted condition were added 1:1 with autologous LCL and incubated with 0 or 10 nM silvestrol for 10 days. Depleted cultures in the absence of silvestrol showed varying outgrowth of LCL; however, silvestrol-treated CoCx all produced a similar loss of LCL. These results indicate that each of these immune cell subsets participates in the anti-tumor activity of the autologous effector population (Supplementary Figure 6).

**Figure 3:**
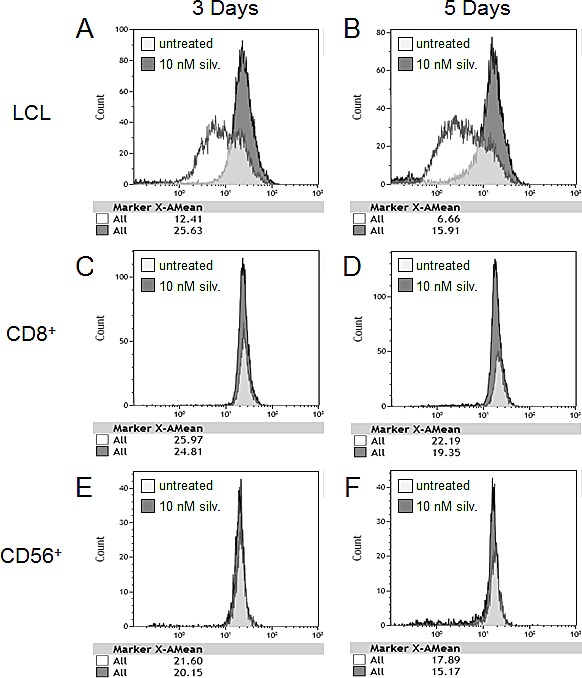
Differential anti-proliferative effect of silvestrol on LCL versus immune effector subsets PBMC or LCL were stained with CFSE. Equal numbers were plated with unstained autologous counterparts (LCL-CFSE with PBMC and LCL with PBMC-CFSE) and then treated with 0 or 10 nM silvestrol for 3 or 5 days. At each time point, CoCx were stained with LIVE/DEAD cell stain and antibodies to CD19 (A, B) for LCL-CFSE CoCx, or CD8 (C, D) or CD56 (E, F) for PBMC-CFSE CoCx. Loss of CFSE mean fluorescence intensity (MFI) indicates an increase in proliferation. Results shown are representative of 3 individual experiments.

To further explore the differential effect of silvestrol on the proliferative capacity of tumor targets and effector populations, LCL or PBMC were stained with the membrane dye CFSE and equal numbers were plated with their unstained autologous counterparts (LCL-CFSE with PBMC, and LCL with PBMC-CFSE). Cultures were treated with 0 or 10 nM silvestrol for 3 or 5 days (Figure 3). LCL-CFSE cultures were then stained for CD19, and PBMC-CFSE cultures were stained for either CD8 or CD56. Gates were set on the subset of interest and analyzed for CFSE intensity (proliferating cells lose CFSE intensity with each cell division). At 3 and 5 days, LCL showed nearly a 50% decrease in proliferation with silvestrol treatment under these CoCx conditions, consistent with growth arrest (Figure 3A, 3B). Conversely, the proliferation rate of both adaptive (CD8+) and innate (CD56+) immune effectors remained unchanged in silvestrol-treated versus untreated CoCx (Figure 3C-3F), as indicated by little or no change in CFSE intensity. These results indicate that silvestrol differentially affects cell proliferation in tumor cells versus immune effector subsets.

### Silvestrol preserves cytotoxic function of adaptive and innate immune effectors

**Figure 4:**
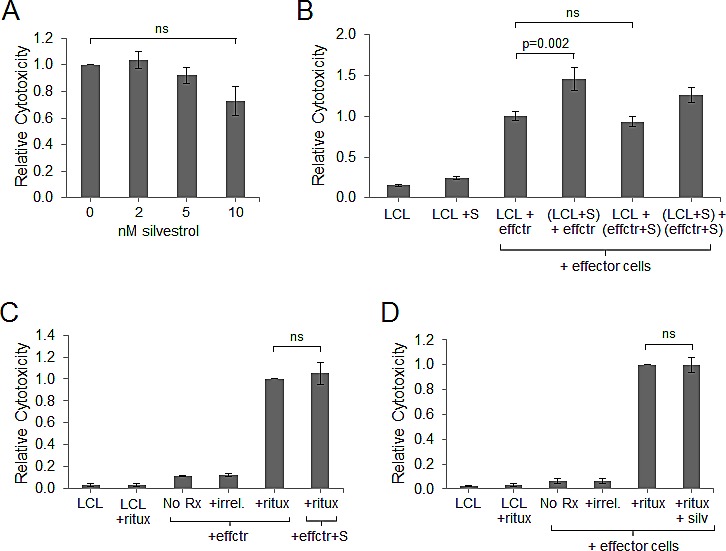
Effector cell cytotoxic activity is maintained in the presence of silvestrol (A) Cytotoxicity assays were performed using autologous effectors expanded 14 days in the presence of irradiated LCL and 10 nM silvestrol. Targets were fresh CFSE-stained LCL at an effector:target (E:T) ratio of 20:1. After a 4 hr incubation, cells were stained with 7-AAD and washed, and cells positive for both CFSE and 7-AAD were enumerated by flow cytometry. Data are shown relative to the vehicle-only control and are the averages of three independent experiments. Bars show mean ± SD. Differences were not significant (ns). (B) Effectors were expanded in the presence of irradiated LCL in the absence of silvestrol. LCL and effectors (effctr) were then incubated separately for 18 hr without or with 10 nM silvestrol (+S). Cytotoxicity assays were then performed as in (A). Data are shown relative to the vehicle-only control and are the averages of three independent experiments. Bars show mean ± SD. (C) Antibody-dependent cell-mediated cytotoxicity (ADCC) assay using NK cells (effctr) from co-cultures grown in the absence or presence of 10 nM silvestrol (+S) mixed with CFSE-stained autologous LCL targets at an E:T ratio of 20:1. Cells were incubated without (No Rx) or with 5 μg/ml rituximab (ritux) or herceptin (irrel). Cytotoxicity was measured by CFSE and 7-AAD dual-positive events. Data are shown relative to the positive control (effectors + targets + ritux, without silvestrol) and are the averages of three individual experiments. Bars show mean ± SD. (D) ADCC assays were performed as in (C), but using fresh non-autologous NK cells incubated 18 hr without or with 10 nM silvestrol (+S). Data are shown relative to the positive control (effectors + targets + ritux) and are the averages of three individual experiments. Bars show mean ± SD.

We next analyzed immune cell function as part of the indirect anti-tumor activity of silvestrol. Viable LCL cells were stained with CFSE, and then incubated at an effector-to-target ratio of 20:1 with autologous PBMC that had been expanded in the presence of irradiated LCL, with or without silvestrol, for 14 days. After a 4 hr incubation, cells were stained with the viability dye 7-AAD and washed. Direct cytotoxic activity of effectors against the CFSE-labeled LCL targets was measured by gating on CFSE-positive events and measuring 7-AAD-positive cells. As shown in Figure 4A, effector cells expanded in the presence of silvestrol largely maintained their cytotoxic activity against autologous LCL compared to effector cells expanded in the absence of silvestrol (N=3; differences between 0 and 10 nM silvestrol were not significant).

Given that LCL cells were depleted and effector cell cytotoxic function was maintained in non-irradiated CoCx, we hypothesized that silvestrol lowers the apoptotic threshold of LCL. To address this, PBMC were co-cultured with irradiated LCL for 14 days. These expanded immune effector cells (comprised of both CD8+ T-cells and CD56^+^ NK cells) and fresh, viable LCL were then incubated separately with silvestrol or vehicle control for 18 hr and washed. Cells were then combined, and after a 4 hr incubation, cytotoxicity assays were performed as described above. Silvestrol-treated effectors showed no decrease in their ability to kill LCL targets compared to untreated effectors (p=0.287; Figure 4B). However, LCL target cells pre-treated with silvestrol were more efficiently killed compared to untreated targets (p=0.002). These data indicate that silvestrol, even at concentrations that show minimal direct cytotoxicity, significantly increases the sensitivity of tumor cells to effector cell-mediated killing.

Next, we utilized ADCC assays to measure the innate immune response of NK cells to LCL tumor targets in the presence of silvestrol. Effector cells were expanded in the presence of irradiated LCL, with or without 10 nM silvestrol, for 14 days prior to enriching for NK cells via negative selection. Fresh CFSE-labeled LCL targets were incubated with rituximab (anti-CD20, expressed on LCL targets) or the negative control antibody herceptin (anti-HER2) in the presence of NK cells. After 4 hr, ADCC was evaluated by gating on CFSE events (stained targets) and analyzing the percentage of 7-AAD positive cells. Rituximab-mediated ADCC of NK cells was similar between effectors expanded with versus without silvestrol (p=0.838; Figure 4C). To determine the acute effect of silvestrol on ADCC, fresh (non-autologous) NK cells were obtained from healthy donors, incubated 18 hr with or without 10 nM silvestrol, then washed and mixed with CFSE-stained LCL. As shown in Figure 4D, silvestrol pre-incubation had no significant effect on the ADCC activity of freshly isolated NK cells (p=0.854).

### Antigen-specific immune responses are maintained in the presence of silvestrol

**Figure 5:**
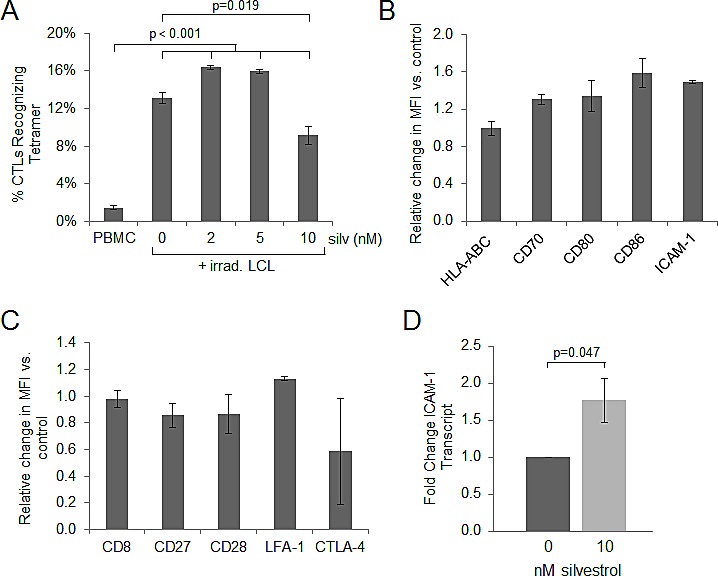
Antigen specific immune responses are maintained in the presence of silvestrol (A) Effectors were expanded 14 days in the presence of irradiated LCL, with or without silvestrol as indicated, using LCL and autologous PBMC from one HLA-B8 donor. A flow cytometric HLA-tetramer assay was used to analyze the ability of antigen-specific CTL to detect EBV BZLF-1 protein bound to MHC class I. The increase in BZLF-1-specific CTL following exposure to irradiated LCL was significant under all conditions (p<0.001 for each 0, 2, 5 and 10 nM silvestrol treatment compared to non-exposed, baseline PBMC), although the expansion with 10 nM silvestrol was less than in the 0 nM (vehicle control) condition (p=0.019). (B) LCL were incubated in 0 or 10 nM silvestrol for 48 hr and analyzed for surface expression of immunological synapse and co-activation proteins. Change in MFI when treated with silvestrol is shown. Bars show mean ± SD from 3 individual donors; differences with silvestrol treatment relative to vehicle were not significant. (C) PBMC from the same 3 donors as in (B) were incubated with 0 or 10 nM silvestrol for 24 hr followed by stimulation with 1 μg/ml anti-hu-CD3 for 18 hr. PBMCs were gated on live, CD8^+^ T-cells, and cognate molecules to those shown in (B) were analyzed for change in MFI with silvestrol treatment. Bars show mean ± SD; differences with silvestrol treatment were not significant. (D) Real-Time RT-PCR analysis of ICAM-1 expression relative to TBP (control gene) in LCL (N=3) incubated 48 hr with 0 or 10 nM silvestrol. Fold change was calculated using the ΔΔCt method [54]. The silvestrol-induced increase in ICAM-1 at the mRNA level was borderline significant (p=0.047).

We next examined the effect of silvestrol on the development of EBV antigen-specific CTL. Effector populations were expanded for 14 days in the presence of irradiated LCL with or without silvestrol. A flow cytometric tetramer assay was used to analyze the ability of antigen-specific CTL to detect the immunodominant HLA-B8-restricted RAK epitope of the EBV lytic protein BZLF-1, as previously described [18]. BZLF-1 has been shown to play an important role in the development of B cell lymphomas, although the precise mechanism remains unclear [38]. The CTL present in baseline PBMC not exposed to irradiated LCL exhibit very low-level detection of EBV BZLF-1 antigen; however, effectors expanded in the presence of irradiated LCL show significant increases in BZLF-1-specific CTL (p<0.001; Figure 5A). Regardless of silvestrol treatment, all PBMC cultures exposed to irradiated LCL showed significant expansion of BZLF-1-specific CD8+ T-cells (p<0.001 for each condition relative to baseline PBMC), although the expansion with 10 nM silvestrol was less than in the untreated (vehicle control) condition (p = 0.019). These results demonstrate that at least at these low concentrations, silvestrol does not block the development of antigen-specific CTL (Figure 5A).

Since effectors expanded in the presence of silvestrol maintain the ability to recognize HLA-presented viral peptide, we next evaluated the expression of immunological synapse proteins on effectors and targets, as modulation of these proteins has been shown as a mechanism for tumor immune escape [39, 40]. First, LCL from 3 donors were incubated for 48 hr with 0 or 10 nM silvestrol. After incubation, LCL were analyzed for MHC class I (HLA-ABC), co-stimulatory molecules (CD70, CD80 and CD86), and integrin-binding protein CD54 (ICAM-1). Incubation with silvestrol did not significantly alter the intensity of expression of class I MHC, nor did it have a significant impact on the expression of co-stimulatory molecules or ICAM-1 on tumor cells (Figure 5B). Next, we analyzed the cognate immunological synapse molecules on the surface of activated immune effector cells. PBMC from 3 donors were incubated with 0 or 10 nM silvestrol for 24 hr followed by overnight stimulation with anti-human CD3. Cells were incubated with labeled antibodies to the molecules of interest and evaluated by flow cytometry, gating on live, CD8^+^ events. Silvestrol did not significantly affect surface levels of CD8 (T-cell co-receptor and MHC class I binding protein), CD27 (co-stimulatory molecule that binds CD70), CD28 (CD80/86 activating binding partner), or LFA-1 (integrin that is bound by ICAM-1 to stabilize the synapse). Although silvestrol treatment decreased expression of the inhibitory molecule CTLA-4 on some CTL, these effects were more variable between samples and thus overall did not reach significance (Figure 5C). As LMP-1 has been shown to induce the transcription of ICAM-1 [41], we analyzed ICAM-1 transcript levels by real-time RT-PCR. Unexpectedly, despite the silvestrol-mediated depletion of LMP-1, ICAM-1 transcript was moderately but significantly increased with silvestrol treatment (p=0.047; Figure 5D). Together, these data demonstrate that silvestrol does not significantly impact activating components of the immunological synapse or co-stimulatory molecules on tumor targets. Importantly, CTL expanded in the presence of silvestrol retain the ability to recognize and proliferate in response to EBV recall antigens presented on LCL tumor cells.

### *In vivo* efficacy of silvestrol in the hu-PBL-SCID model of EBV-LPD

**Figure 6:**
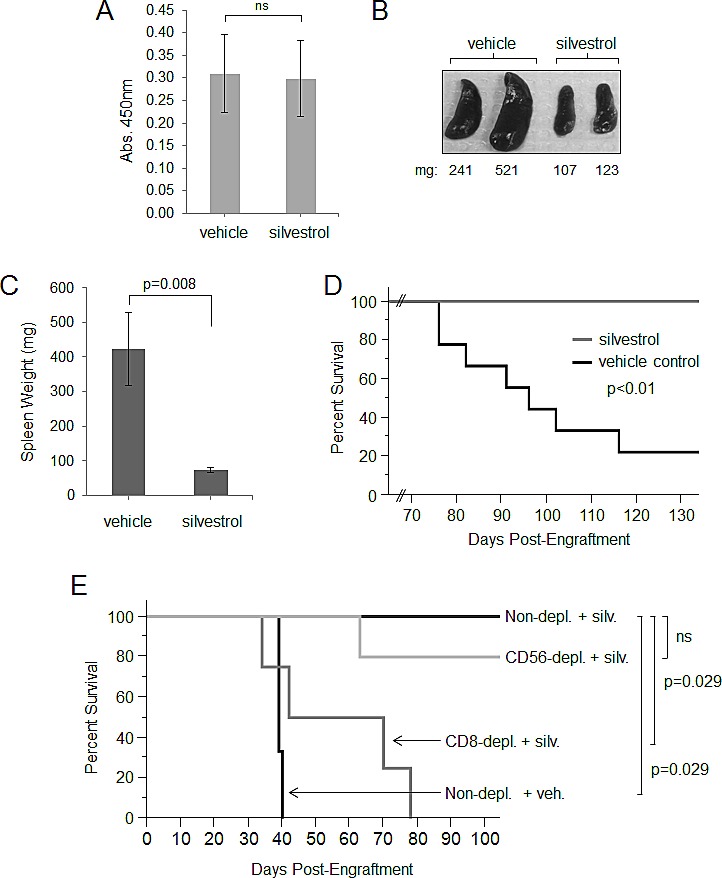
Evaluation of *in vivo* therapeutic activity of silvestrol in hu-PBL SCID model SCID mice depleted of murine NK cells were injected intraperitoneally with 5×10^7^ PBMC from a healthy EBV-seropositive human donor. Treatment with vehicle or 1.5 mg/kg silvestrol (N=14 per group) every other day by the IP route began two weeks post-engraftment. (A) Human Ig ELISA on peripheral blood from all mice (N=28) 4 weeks after engraftment; averages for both groups are shown. Bars show mean ± SD. Differences were not significant (ns). (B) Spleens are shown from two mice from each group 8 weeks post-engraftment, with weights in mg. (C) Spleen weights were recorded from all mice upon euthanasia or end of study (day 140 post-engraftment) (N=9 for vehicle control and N=7 for silvestrol group). Bars show mean ± SD; differences were significant (p=0.008). (D) Kaplan-Meier analysis of overall survival (vehicle control N=9; silvestrol-treated N=7; p<0.01). (E) Specific lymphocyte subsets were depleted from PBMC directly prior to engraftment using immunomagnetic bead depletion for CD8 (cytotoxic T cells), CD14 (monocytes), or CD56 (NK cells). Biotin-only conjugated beads were used for the control (mock-depleted) condition. Efficiency of depletion was verified by flow cytometric analyses and show to be greater than 90%. Equivalent numbers of depleted PBMC preparations (5×10^7^ cells in the mock-depleted condition) were engrafted by intraperitoneal injection into murine NK cell-depleted SCID mice (N=5 per group). After 4 weeks, human Ig levels were assessed by ELISA, and only mice showing engraftment by this parameter were included in the study. Kaplan-Meier analysis of overall survival was then performed. The differences between the non-depleted vehicle control (N=3) vs. non-depleted silvestrol-treated (N=4) groups, and between the CD8-depleted silvestrol-treated vs. non-depleted silvestrol-treated groups (N=4 each), were significant (p=0.029 each). The difference between CD56-depleted silvestrol-treated (N=5) vs. non-depleted silvestrol-treated (N=4) was not significant. Also, there was no difference between CD8-depleted vs. non-depleted vehicle controls (not shown).

The hu-PBL SCID model has been used to identify experimental therapeutic strategies to prevent or treat spontaneous EBV-LPD [15, 16, 42-45]. PBMC from a healthy EBV-positive donor were injected into a group of SCID mice that had been pretreated with anti-asialo (GM1) to deplete murine NK cells. Mice were randomized (N=14/group), and IP treatments with vehicle or silvestrol (1.5 mg/kg) every 48 hr began 2 weeks after engraftment. Two weeks after treatment began (4 weeks post-engraftment), an ELISA was performed to evaluate human IgG plasma levels. All 28 mice were shown to produce human IgG (Figure 6A), with no significant difference between the silvestrol-treated versus vehicle-treated groups. This finding not only confirmed successful engraftment, but also showed that silvestrol did not adversely affect production of human IgG by xenografted B-lymphocytes, suggesting that silvestrol also may allow the preservation of adaptive humoral immune responsiveness. At 4 and 8 weeks post-engraftment, flow cytometry analysis (hu-CD45^+^/CD19^+^/CD3^−^) was performed on spleen cells from 2 mice in each group. Mice in the vehicle control group showed substantial tumor infiltration of the spleen without obvious nodal involvement, whereas no tumor cells could be detected in the spleens of mice from the silvestrol-treated group (not shown). By 8 weeks post-engraftment, spleens from mice in the control group showed profound splenomegaly characterized by human B-cell infiltration, while spleens from silvestrol-treated animals exhibited normal spleen mass (Figure 6B). Spleens from both treatment groups were also obtained from each animal as euthanasia criteria were met, or for the silvestrol-treated mice, at the end of study (140 days). Mice in the vehicle control group showed significantly enlarged spleens relative to silvestrol-treated mice (p<0.01; Figure 6C). Finally, silvestrol significantly prolonged survival compared to vehicle (7/7 silvestrol-treated mice vs. 2/9 control mice alive at day 140, p<0.004; Figure 6D). At the end of the study, all animals were examined for presence of EBV-LPD by necropsy as well as flow cytometric evaluation of spleen cells. While the remaining vehicle-treated mice exhibited substantial tumor infiltration of the spleen, no such lymphocyte infiltration could be detected in the spleens of any of the silvestrol-treated mice, nor did these animals exhibit any other obvious signs of lymphoma upon necropsy (not shown). We also evaluated normal blood cell populations following silvestrol treatment (1.5 mg/kg IP every other day for 28 days) in healthy, immune-competent C57BL/6 mice. No significant changes were found in total leukocyte, lymphocyte, and erythrocyte subsets, although a moderate (approximately 1.4 fold) increase in platelets was observed (Supplementary Figure 7A). Total spleen cells from these healthy mice were also investigated for effects on normal lymphocyte subsets; no differences were found with silvestrol treatment on murine CD4+ or CD8+ (T-cell), CD19+ (B-cell) or NK1.1+ (NK cell) subsets.

### CD8+ T lymphocytes are necessary for *in vivo* efficacy of silvestrol

To further investigate the importance of immune effector cells in silvestrol's dramatic protective effect in EBV-LPD, the previous *in vivo* experiment was repeated with prior depletion of specific human lymphocyte subsets. Before engraftment, PBMC were subjected to immunomagnetic bead depletion for cells carrying CD8 (CTL), CD14 (monocytes), or CD56 (NK cells). Mock depletion was performed using biotin-conjugated beads as a negative control. Efficiency of depletion was verified by flow cytometric analyses to be greater than 90% (Supplementary Figure 8). A total of 5×10^7^ mock-depleted PBMC, or equivalent percentages from each of the subset depletions as described previously [18], were engrafted into SCID mice depleted of murine NK cells by anti-asialo injections (N=5/group). As with the prior experiment, IP treatment with 1.5 mg/kg silvestrol or vehicle every 48 hr began 2 weeks after engraftment. Prior experience with this model shows PBMC depleted of CD3/CD8+, CD56+, or CD14+ cells do not affect human cell engraftment and production of human IgG levels [16]. Two weeks after treatment began (4 weeks post-engraftment), an ELISA was performed to evaluate human IgG. Only mice shown to be engrafted by production of human IgG were used in the study. The mock-depleted mice showed results similar to the previous experiment, with 0/3 surviving in the vehicle group and 4/4 surviving in the silvestrol-treated group (p=0.029) (Figure 6E). Notably, when CD8^+^ T-cells were depleted, silvestrol no longer provided effective anti-tumor protection (0/4 surviving in silvestrol-treated CD8-depleted group versus 4/4 in the silvestrol-treated mock-depleted group; p=0.029), nor did it reduce tumor burden as measured by spleen mass (not shown). Mice receiving CD14+ (monocyte) or CD56+ (NK cell) depleted engraftments exhibited an intermediate response to silvestrol treatment (2/4 and 4/5 surviving, respectively) that did not reach statistical significance with this number of animals. These data indicate that silvestrol is indirectly mediating protection through immune surveillance mechanisms, and that at least CD8^+^ T-lymphocytes are essential in the silvestrol-mediated clearance of tumor.

## DISCUSSION

EBV is associated with a broad spectrum of benign and malignant diseases that, collectively, represent a growing number of cases in immunocompromised individuals and in our aging population [46-48]. Current therapies for EBV-LPD often lead to profound immune suppression and subsequent development of life-threatening opportunistic infections and EBV reactivation. Thus, an ideal agent to treat patients with EBV-LPD would possess direct anti-tumor activity while preserving the host anti-tumor immune function. Previous studies showed the direct anti-tumor activity of the translation inhibitor silvestrol in leukemia and lymphoma [3, 6, 11-14], and selective cytotoxicity to malignant B-cells compared to normal lymphocytes [13]. This activity appears to be due to the loss of short half-life protective proteins resulting from direct translation inhibition. Here we show that, in addition to this activity, silvestrol is also an immune-preserving agent. We provide a characterization of the unique anti-tumor and immune-potentiating properties of this drug using *in vitro* and *in vivo* models of EBV-LPD.

Our principle findings show that direct anti-proliferative activity of silvestrol toward LCL at physiologically achievable concentrations is moderate. However, these low concentrations of silvestrol, when added to co-cultures of LCL and autologous PBMC, produce complete ablation of the malignant LCL cells. Importantly, the *in vivo* EBV-LPD model revealed a remarkable survival advantage with silvestrol treatment, far greater than we and others have previously shown with silvestrol using xenografts in immune deficient mice [13, 14]. Together, these results indicate that the indirect effects of silvestrol on tumor cells via innate and adaptive immune components may be at least as important as its direct effects. As most *in vivo* studies with silvestrol to date have used tumor xenografts in immune deficient mice, this crucial aspect was previously unknown.

The direct growth inhibitory activity of silvestrol as well its ability to sensitize LCL to immune-mediated killing appears to be associated with a loss of the LMP-1 oncoprotein. Drugs and cytotoxic T-cell preparations directly targeting LMP-1 have been shown to prevent metastasis, promote apoptosis and enhance radiosensitivity, identifying LMP-1 as a potential therapeutic target for EBV-driven malignancies [26-30]. The ablation of LMP-1 protein has been shown to interfere with several downstream signaling pathways including AKT [28] as well as STAT1 and STAT3 [34, 36, 49], and our results support these findings. Inhibition of these pathways may lower tumor apoptotic threshold and allow sensitization to effector immune cell anti-tumor activity [6, 11]. Certainly, we observed that low doses of silvestrol enhanced sensitivity of LCL tumor targets to innate and adaptive immune effector-mediated killing while producing little to no effect on those effector cells' functional capabilities. We are presently investigating the mechanism accounting for this reduced apoptotic threshold in LCL. It was previously reported that the classical chemotherapeutic agents paclitaxel, cisplatin, and doxorubicin sensitize several types of solid tumor cells to cytotoxic T-lymphocytes by increasing tumor cell permeability to granzyme B [50].

It has been previously reported that silvestrol sensitizes tumor cells to classical chemotherapeutic agents including doxorubicin [6, 11]. However, as standard chemotherapies used in the treatment of lymphoma impair adaptive and innate cell-mediated immunity, such combinations may potentially circumvent the immune-preserving advantage of silvestrol. Here we describe an entirely new characteristic of silvestrol, which is the maintenance of adaptive immune effectors (EBV-specific CTL) and innate immune effectors (NK cells) using concentrations that have direct, albeit moderate, anti-tumor activity. These studies employed an established *in vitro* model in which EBV-driven lymphoma cells (LCL) are incubated with autologous PBMC. Left untreated, the LCL ultimately outpace the expanding effector populations, and overtake the culture. With the addition of as little as 10 nM silvestrol, we observed complete ablation of viable LCL in these autologous co-cultures while effector cell populations (*i.e.* CD8+ CTL and CD56+ NK cells) were maintained; importantly, this effect of silvestrol on LCL is greatly reduced in the absence of effector cells. Furthermore, proliferation assays in silvestrol-treated co-cultures show a substantial decrease in proliferation of LCL targets without a corresponding change in proliferation of effector cells. We are currently investigating the differential activity of silvestrol on cell cycle checkpoints in tumor versus immune effector populations.

Besides affecting absolute cell numbers, classical chemotherapy is known to have a deleterious effect on cancer-relevant immune cell function including NK cell-mediated ADCC [51, 52]. Our experiments show that silvestrol does not significantly impact NK cell ADCC activity, either when NK cells were expanded in the presence of silvestrol or when silvestrol was added immediately prior to the ADCC assay. Additionally, EBV proteins also mediate multiple effects to evade adaptive immune detection of virally-infected cells, and appropriate adaptive immune recognition of EBV proteins by CTL is essential to avoid development of EBV-related malignancies [53]. CTL exposed to silvestrol retained the ability to recognize presented viral peptide in the context of class I MHC, and showed little or no change in cytotoxic function or in expression of immune synapse components. As silvestrol treatment does not impede the expansion of EBV-specific CTL or alter the expression of immune synapse or co-stimulatory molecules, this may explain how silvestrol treatment can preserve immunological responsiveness against EBV-LPD.

Our *in vivo* studies using the hu-PBL SCID model provide significant insight into the efficacy of silvestrol in treating EBV-LPD. Importantly, our *in vivo* experiment using grafts depleted of effector cell subsets establishes that CD8+ CTL are necessary for full silvestrol efficacy in this model. The moderate loss of protection when other lymphocyte subsets are depleted suggests that silvestrol is sensitizing tumor cells for clearance through other immune effectors as well, although a larger study is required to address this. Furthermore, *in vivo* experiments using animals with intact immune systems did not show significant alteration in total lymphocytes or modulation in lymphocyte populations with silvestrol treatment, consistent with previously reported data [12]. Together, these studies provide further evidence of silvestrol's limited toxicity to immune effectors.

In summary, we present new information regarding the efficacy of the translation inhibitor silvestrol in EBV-LPD and demonstrate that this efficacy is largely due to the maintenance of number and function of both innate and adaptive anti-tumor immune components. Additionally, silvestrol promotes modest direct anti-tumor activity against EBV-transformed lymphoma cells, likely by depleting the oncogenic viral protein LMP-1 and interfering with downstream survival pathways. Other chemotherapeutic regimens currently in use for the treatment of EBV-LPD display a non-specific cytotoxicity both to the tumor target as well as immune effectors, leading to elimination of potent memory anti-tumor responses. This effect leaves patients vulnerable to disease relapse and at increased risk of serious and potentially lethal opportunistic infections. The ability of silvestrol to simultaneously exert direct anti-proliferative activity against EBV-transformed tumor cells while sparing adaptive and innate immune effector function is highly unusual if not unique, and shows promise for future clinical translation.

## METHODS

### Ethics statement

Investigations were conducted in accordance with the ethical standards and according to the Declaration of Helsinki and according to national and international guidelines, and were approved by the OSU institutional review board.

### Reagents

Silvestrol was isolated as described [3]. The active metabolite of fludarabine, 2-fluoro-ara-A, was obtained from Sigma (St. Louis, MO).

### Cells and cultures

EBV-transformed lymphoblastoid cell lines (LCL) were derived *in vivo* by engrafting severe combined immune-deficient (SCID) mice (Taconic, Hudson, NY) with human peripheral blood mononuclear cells (PBMC) from healthy EBV-positive donors [15, 16]. Co-cultures (CoCx) were created in 96-well plates by mixing LCL (either non-irradiated or irradiated with 14,000 rad) with equal numbers of autologous PBMC. Cultures were grown in the presence of 10 U/ml interleukin-2 (IL-2; Prometheus, San Diego, CA), and were given a single dose of silvestrol (10 nM) before being cultured for 10-14 days.

### Immunoblot analysis

LCL were subjected to immunoblotting as described [17]. Antibodies were from Cell Signaling Technology (Danvers, MA) (β-actin, pSTAT1, pSTAT3, STAT1, STAT3, pAkt, or Akt) or DakoCytomation (Carpinteria, CA) (LMP-1).

### Proliferation assays

MTS assays to measure mitochondrial function were performed using the CellTiter96^TM^ assay (Promega, Madison, WI).

### Flow cytometric analyses

Cells were co-stained with LIVE/DEAD reagent (Invitrogen, Grand Island, NY) and hu-CD3-APC, as well as hu-CD4-PE, hu-CD8-PE, hu-CD19-PE (BD Biosciences, San Diego, CA), or hu-CD56-PE (Beckman Coulter, Brea, CA). Events were gathered by gating on cells negative for the LIVE/DEAD stain on an FC500 cytometer (Beckman-Coulter). Cell viability also was measured by flow cytometry using annexin-V-FITC and propidium iodide (PI) (BD Biosciences). Human HLA-B8 tetramers complexed with immunodominant peptide from BZLF-1 (RAKFKQLL) and conjugated with allophycocyanin (APC) [18] were provided by the NIAID Tetramer Facility and the NIH AIDS Research and Reference Reagent Program (Atlanta, Georgia). Where indicated, cell counting beads were used to obtain cell numbers according to manufacturer's description (BD Biosciences).

### Cytotoxicity assays

Non-radioactive flow cytometry-based cytotoxicity assays were performed as described [19, 20]. CoCx (PBMC plus irradiated LCL) were incubated in the presence or absence of silvestrol (10 nM) for 14 days. Fresh autologous LCL cells were stained with carboxyfluorescein diacetate succinimidyl ester (CFSE; CellTrace^TM^, Invitrogen) prior to being mixed with effectors from the irradiated CoCx at an effector:target ratio of 20:1. Cells were stained with the viability dye 7-aminoactinomycin D (7-AAD) (BD Biosciences) and washed. Cytotoxicity was measured by gating on CFSE-positive events and measuring 7-AAD-positive cells. For antibody-dependent cell-mediated cytotoxicity (ADCC) assays, co-cultured effectors were natural killer (NK) cell-enriched and were incubated in the presence or absence of 5 μg/ml rituximab or the irrelevant control antibody herceptin (Genentech, South San Francisco, CA).

### Proliferation assays

CFSE-stained PBMCs were incubated with unstained autologous LCLs, and CFSE-stained LCLs were incubated with autologous unstained PBMCs. At days 3 and 5, cells were stained with anti-CD19-APC for CFSE-LCL, or anti-CD56-APC or anti-CD8-APC for CFSE-PBMC, and analyzed by flow cytometry.

### *In vivo* studies

The Hu-PBL-SCID model has been described [15, 16, 21]. PBMC were obtained from healthy EBV-seropositive donors under an Ohio State University Institutional Review Board-approved protocol. PBMC were injected intraperitoneally (IP) into SCID mice depleted of murine NK cells by pretreatment (plus weekly re-treatment) with anti-asialo (GM1) (Wako, Richmond, VA). Engraftment was confirmed by hu-IgG ELISA [16]. Treatments with vehicle (30% hydroxypropyl-β-cyclodextrin; CTD Holdings, Inc., Alachua, FL) or silvestrol (1.5 mg/kg every 48 hr IP) began 2 weeks post-engraftment.

### Quantitative Reverse Transcription PCR

ICAM-1 (CD54) mRNA levels were determined using the Viia7 Real-Time PCR System (Applied Biosystems, Foster City, CA), using TaqMan Gene Expression Assays for ICAM-1 and TBP (control) (Applied Biosystems).

### Statistics

To account for correlations among observations from the same donor, linear mixed effects models were used to estimate the effect of silvestrol on apoptosis and cell proliferation. Similarly, differences in rituximab-mediated NK cell ADCC activity in effectors expanded in the presence versus absence of silvestrol were examined using mixed effects models. Differences in cell numbers of each of the four subsets (CD3-/CD19+, CD3+/CD8+, CD3-/CD56+, and CD3+/CD4+) were compared between cells incubated with versus without silvestrol using paired t-tests. Differences in surface expression of immunological synapse proteins in activated effectors incubated with versus without silvestrol were also examined using paired t-tests, with an adjusted significance level of 0.01 to control overall Type I error. The proportions of mice alive at end of study were compared between the vehicle control and silvestrol-treated groups using Fisher's exact test. Finally, spleen size differences between silvestrol-treated and control mice were assessed using a two-sample t-test. All analyses were performed using SAS/STAT software version 9.2 (SAS Institute Inc., Cary, NC).

## SUPPLEMENTARY MATERIAL, FIGURES


