# Supplementary material for: The translation inhibitor silvestrol exhibits direct anti-tumor activity while preserving innate and adaptive immunity against EBV-driven lymphoproliferative disease
Source: Oncotarget. 2014 Jun 12;6(5):2693–708. doi: 10.18632/oncotarget.2098 (PMC4413611; doi:10.18632/oncotarget.2098)
Supplement: Supplementary file 1 [file oncotarget-06-2693-s001.pdf]

The translation inhibitor silvestrol exhibits direct anti-tumor activity while preserving innate and adaptive immunity against EBV-Driven lymphoproliferative disease

SUPPLEMENTAL MATERIAL

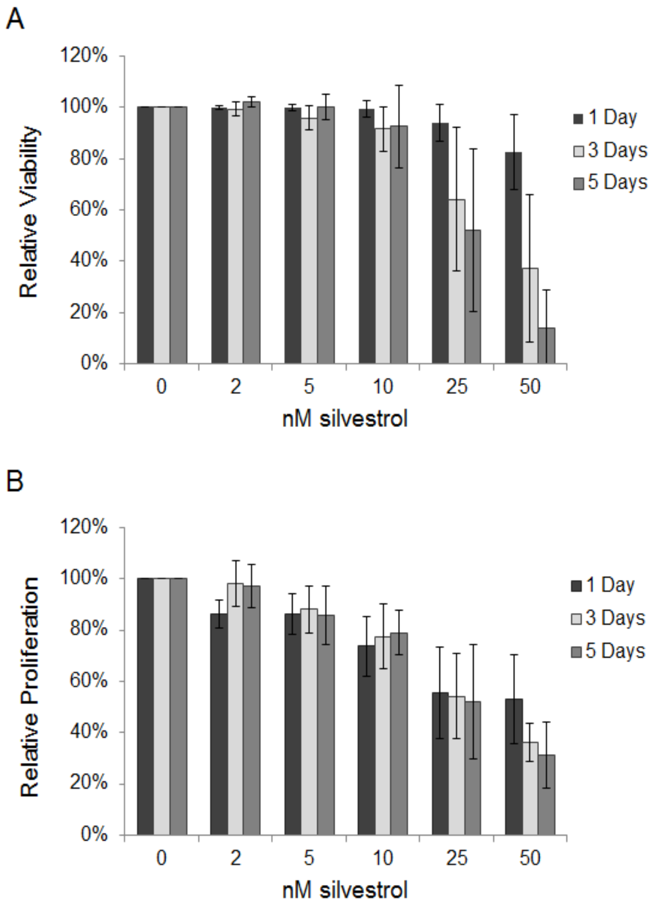

Supplemental Figure 1: Silvestrol promotes direct anti-tumor activity in lymphoblastoid cell lines (LCL). *In vivo*-derived LCL (N=6) were incubated with indicated concentrations of silvestrol or vehicle for 24, 72, or 120 hr. (A) Viability was examined by annexin/propidium iodide (PI) flow cytometry. Data are shown as percent annexin-negative and PI-negative cells relative to the time-matched vehicle control group. Bars show mean ± standard deviation (SD) Differences with silvestrol treatments were significant ($p<0.001$). (B) Proliferation was measured by the MTS assay. Data are shown relative to the vehicle control at each time point. Bars show mean ± SD. Differences with silvestrol treatments were significant ($p=0.006$).

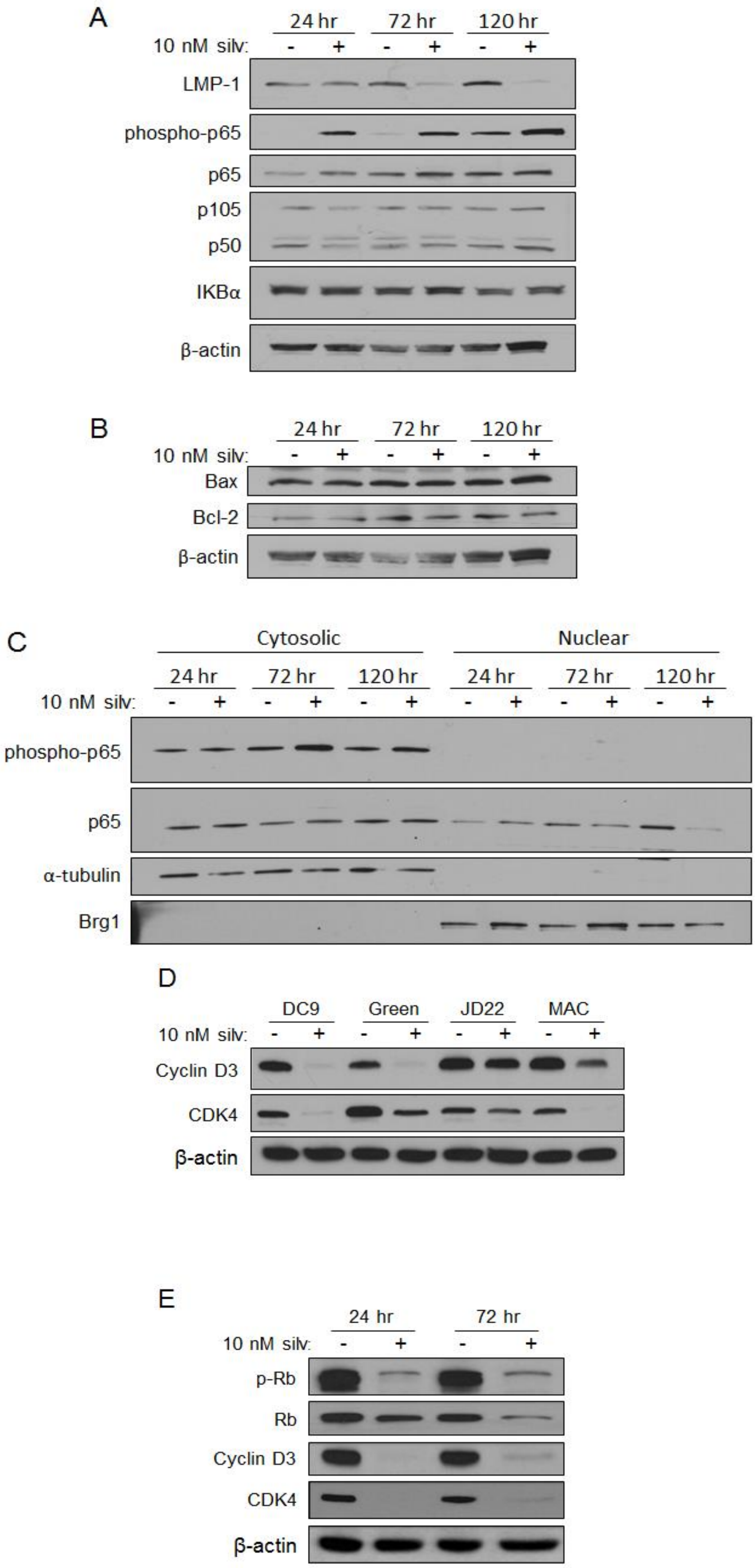

Supplemental Figure 2: Silvestrol modulates LMP-1 and downstream targets. LCL were incubated 24, 72, and 120 hr in the presence of 10 nM silvestrol or vehicle. Whole cell lysates

were immunoblotted for: (A) LMP-1, phosphorylated and total p65, p105/p50, and IKBα; (B) Bax and Bcl-2. β-actin was included as a loading control. Results are representative of four individual LCL. (C) Cytoplasmic and nuclear extracts were prepared from LCL incubated 24, 72, and 120 hr in the presence of 10 nM silvestrol or vehicle and immunoblotted for phosphorylated and total p65. α-tubulin and Brg1 were included as cytoplasmic and nuclear controls, respectively. Results are representative of four individual LCL. (D) Lysates from four LCL incubated 24 hr with vehicle or 10 nM silvestrol were immunoblotted for cyclin D3, CDK4 and β-actin. (D) A representative LCL was incubated 24 and 72 hr with vehicle or 10 nM silvestrol, and lysates were immunoblotted for phosphorylated and total Rb, cyclin D3 and CDK4. β-actin was included as a loading control.

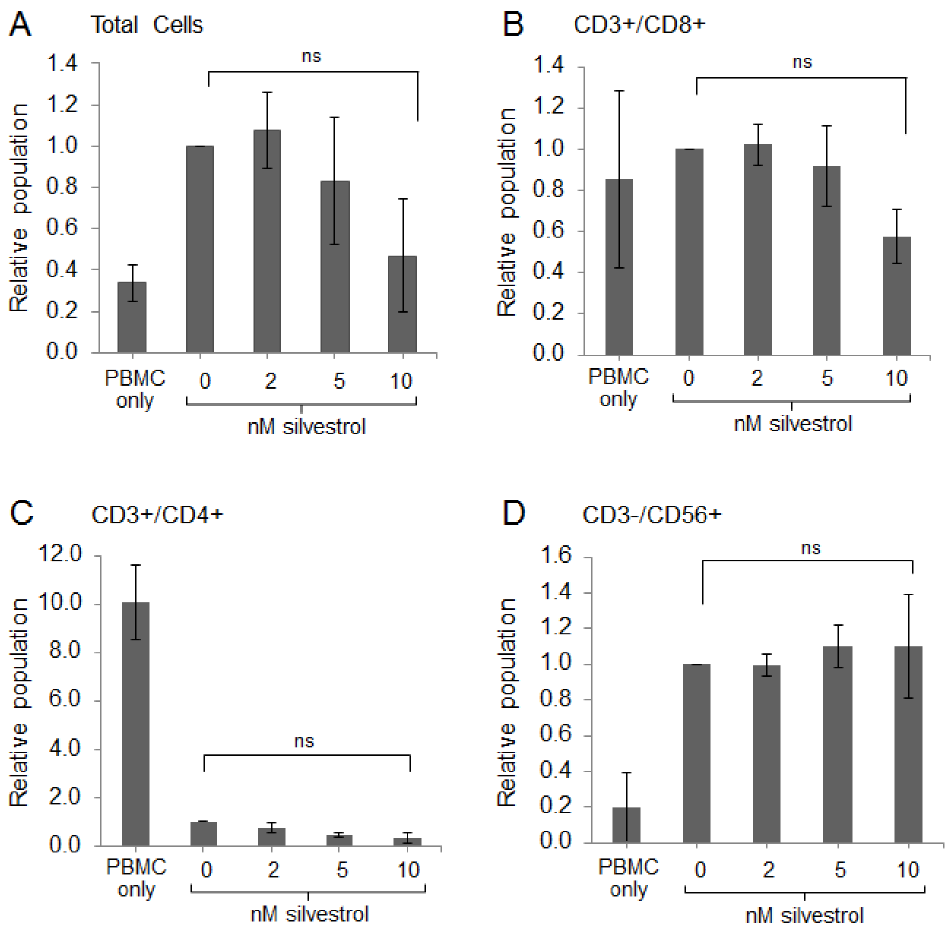

Supplemental Figure 3: T-cell and NK cell populations are maintained in the presence of silvestrol in irradiated LCL co-cultures. CoCx were created by mixing irradiated LCL with equal numbers of autologous PBMC. CoCx (or PBMC alone) were incubated in the presence of 10 U/ml IL-2, and given a single dose of 0 (vehicle only), 2, 5, or 10 nM silvestrol. Flow *cytometric* analysis was conducted on day 14. Live cells were gathered by gating on cells negative for the LIVE/DEAD stain. Data are expressed as percentage of viable population, relative to the vehicle CoCx condition, for: (A) total cells; (B) $CD3^+/CD8^+$ cells; (C) $CD3^+/CD4^+$ cells; (D) $CD3^-/CD56^+$ cells. Results shown are the averages from three individual CoCx; bars show ± SD.

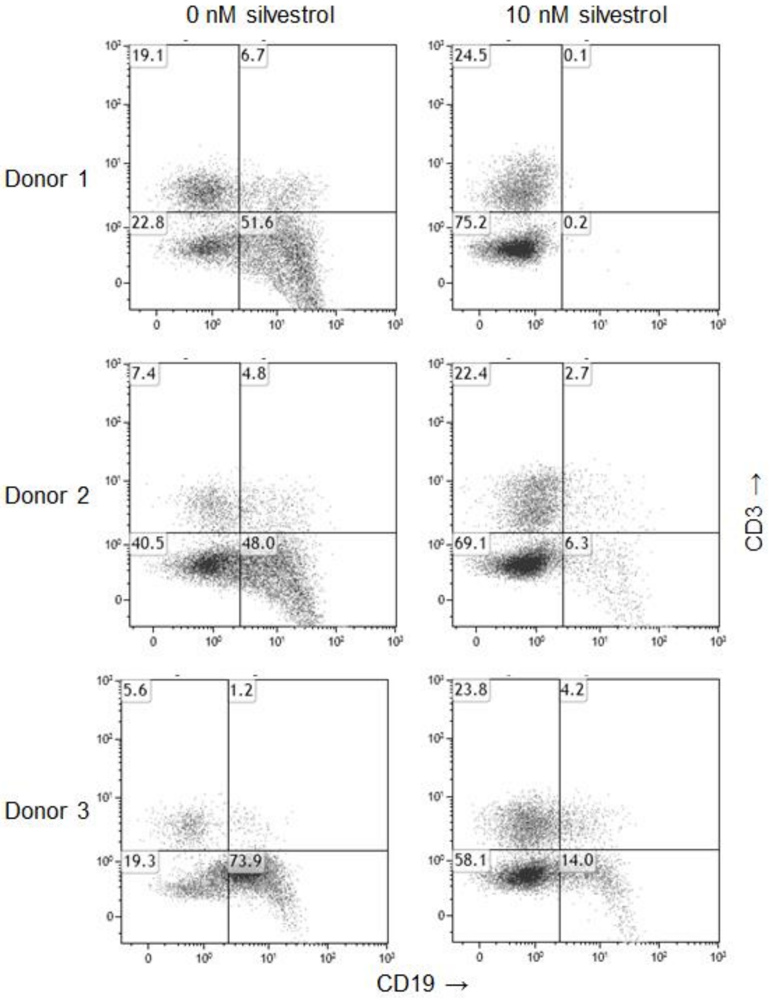

Supplemental Figure 4: Silvestrol leads to loss of non-irradiated LCL in co-cultures from multiple donors. CoCx were created by mixing non-irradiated LCL from three individual EBV-positive donors with equal numbers of autologous PBMC. CoCx, LCL, or PBMC alone were incubated in the presence of 10 U/ml IL-2 and given a single dose of 0 (vehicle only) or 10 nM silvestrol. Flow *cytometric* analysis was conducted on day 10. Cells were stained for CD3 (y-axis) and CD19 (x-axis) and gated on live events (cells negative for the LIVE/DEAD stain). LCL ($CD3^-$/$CD19^+$) are shown in the bottom right quadrant.

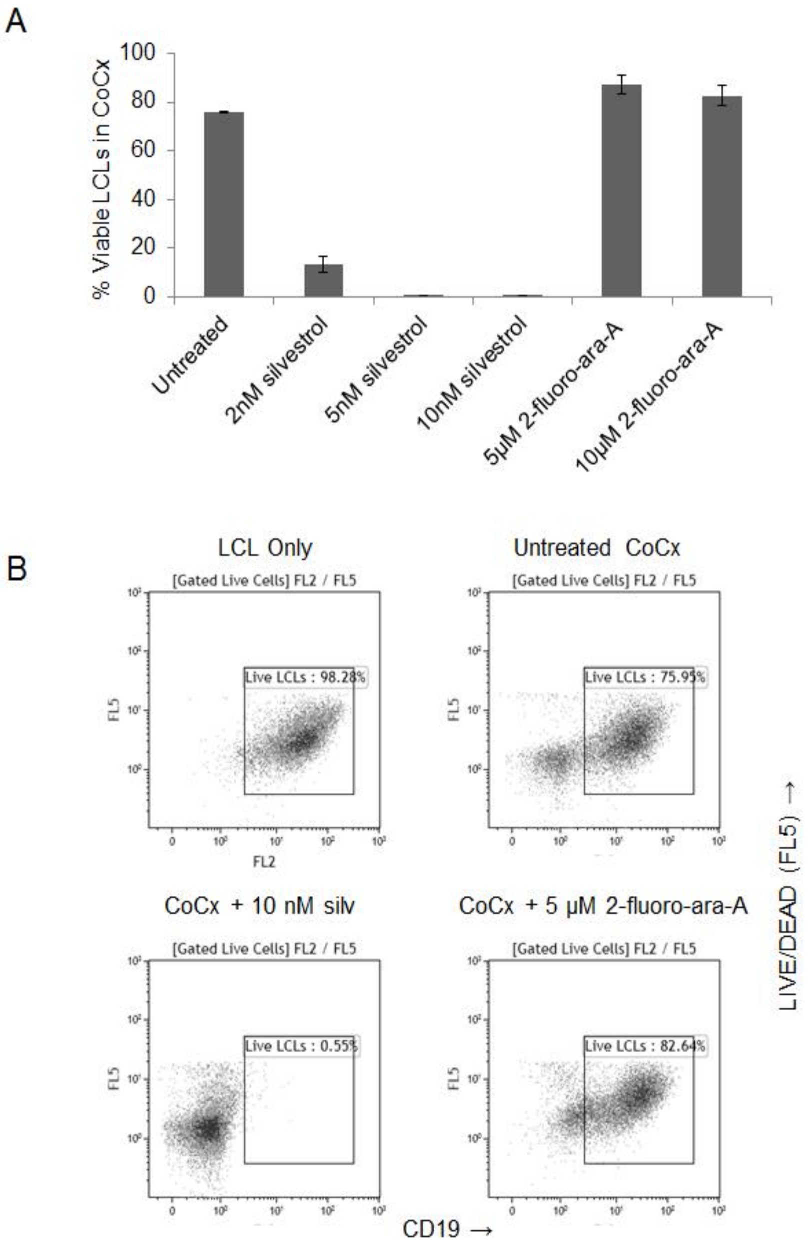

Supplemental Figure 5: Fludarabine does not control outgrowth of LCL. CoCx (N=3) were created by mixing non-irradiated LCL from with equal numbers of autologous PBMC. CoCx or PBMC alone were incubated in the presence of 10 U/ml IL-2 and given a single dose of 0, 2, 5 or 10 nM silvestrol, or 5 or 10 µM 2-fluoro-ara-A (active metabolite of fludarabine). Flow *cytometric* analysis was conducted on day 10 to assess viable cells (*i.e.* cells negative for the LIVE/DEAD stain). (A) Data are expressed as percentage of $CD3^-/CD19^+$ (LCL) in the total viable population. Bars show mean ± SD. (B) Dot plots showing viable LCL (CD19-positive cells negative for LIVE/DEAD stain (FL5)).

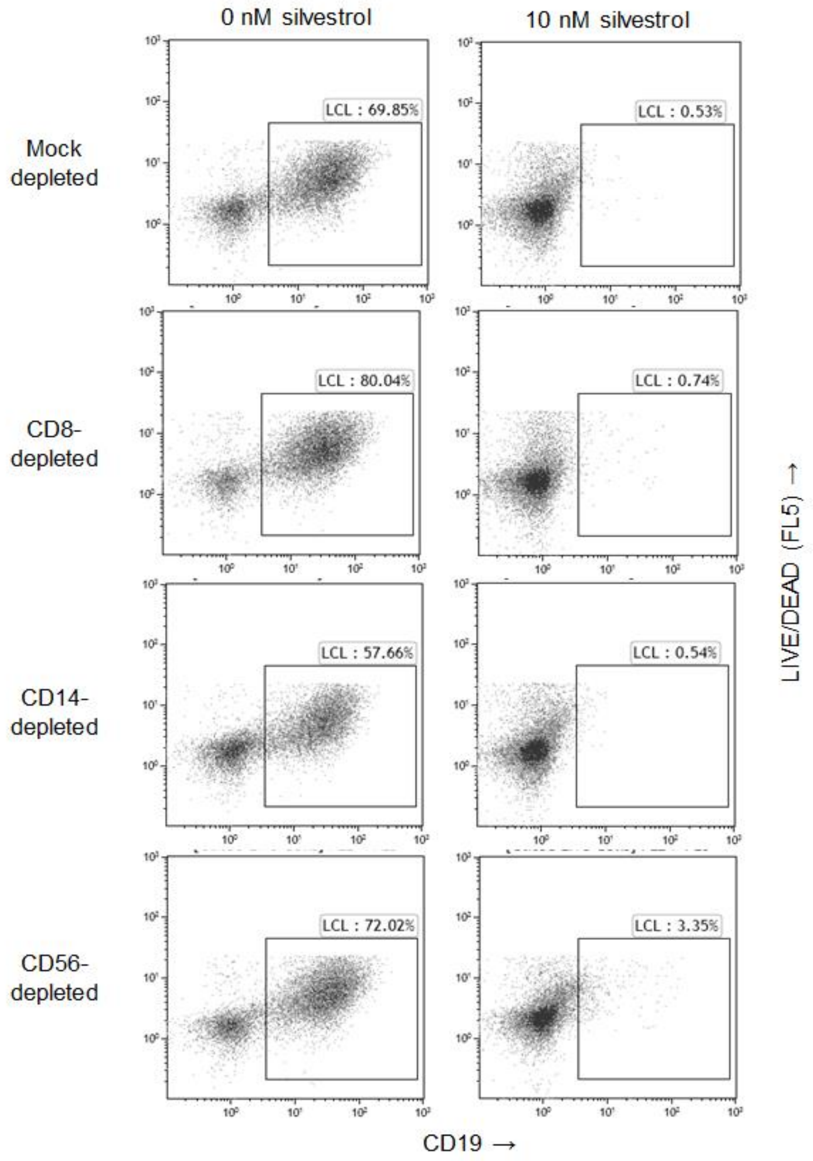

Supplemental Figure 6: Depleted effector subsets still allow for ablation of LCL in silvestrol-treated CoCx. PBMC were subjected to immunomagnetic bead depletion (negative selection) for: CD8 (cytotoxic T cells), CD14 (monocytes) or CD56 (NK cells). Biotin-only conjugated beads were used for the control (mock-depleted). Efficiency of depletion was verified to be greater than 90% by flow cytometry. CoCx were created by mixing non-irradiated LCL from three individual EBV-positive donors with equal volumes of autologous PBMC from each depletion condition. CoCx were incubated in the presence of 10 U/ml of IL-2 and given a single dose of 0 (vehicle only) or 10 nM silvestrol. Flow *cytometric* analysis was conducted on day 10. Live LCL were assessed by gating on CD19+ cells negative for the LIVE/DEAD stain.

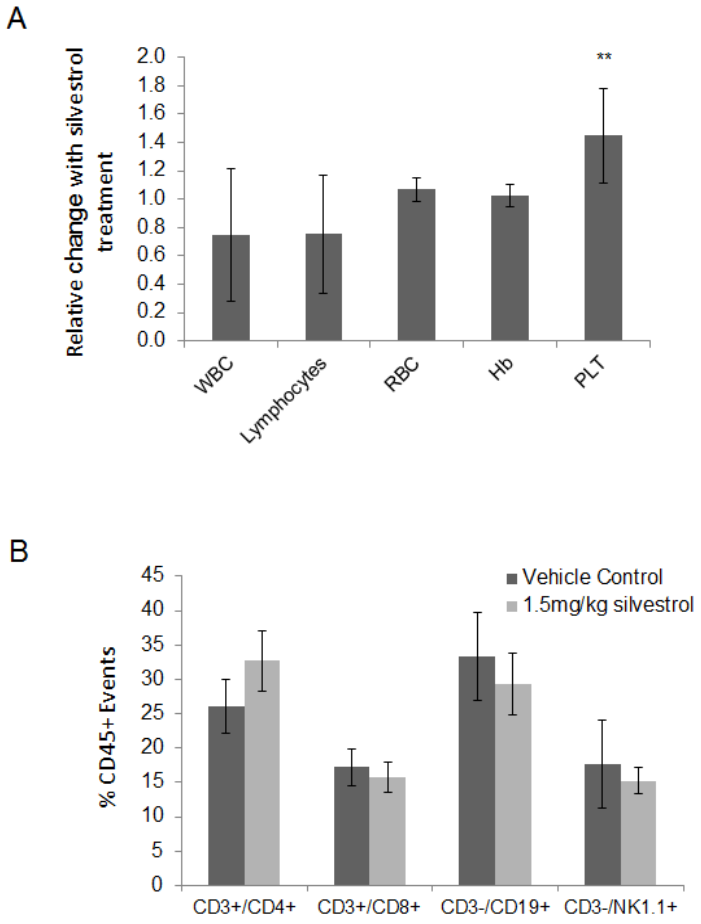

Supplemental Figure 7: Silvestrol shows limited effects on blood cells *in vivo*. Six-week-old C57BL/6 mice were treated with vehicle or 1.5 mg/kg silvestrol IP (N=7 per group) every 48 hr for 28 days. (A) At the end of the trial, complete blood counts were performed to evaluate major circulating populations of blood cells. (WBC = total white blood cells; RBC = red blood cells; Hb = hemoglobin). Data are shown as changes with silvestrol treatment relative to vehicle controls; bars show mean ± SD. The moderate elevation in platelets (PLT) was significant (**$p < 0.01$). (B) Lymphocytes were evaluated in peripheral blood from these mice by gating on murine $CD45^+$ events and analyzing lymphocyte subsets: $C3^+/CD4^+$ (helper T-cells), $CD3^+/CD8^+$ (CTL), $CD3^-/CD19^+$ (B-cells) and $CD3^-/NK1.1^+$ (NK cells). Bars show mean ± SD. No significant changes were observed in any of the lymphocyte populations.

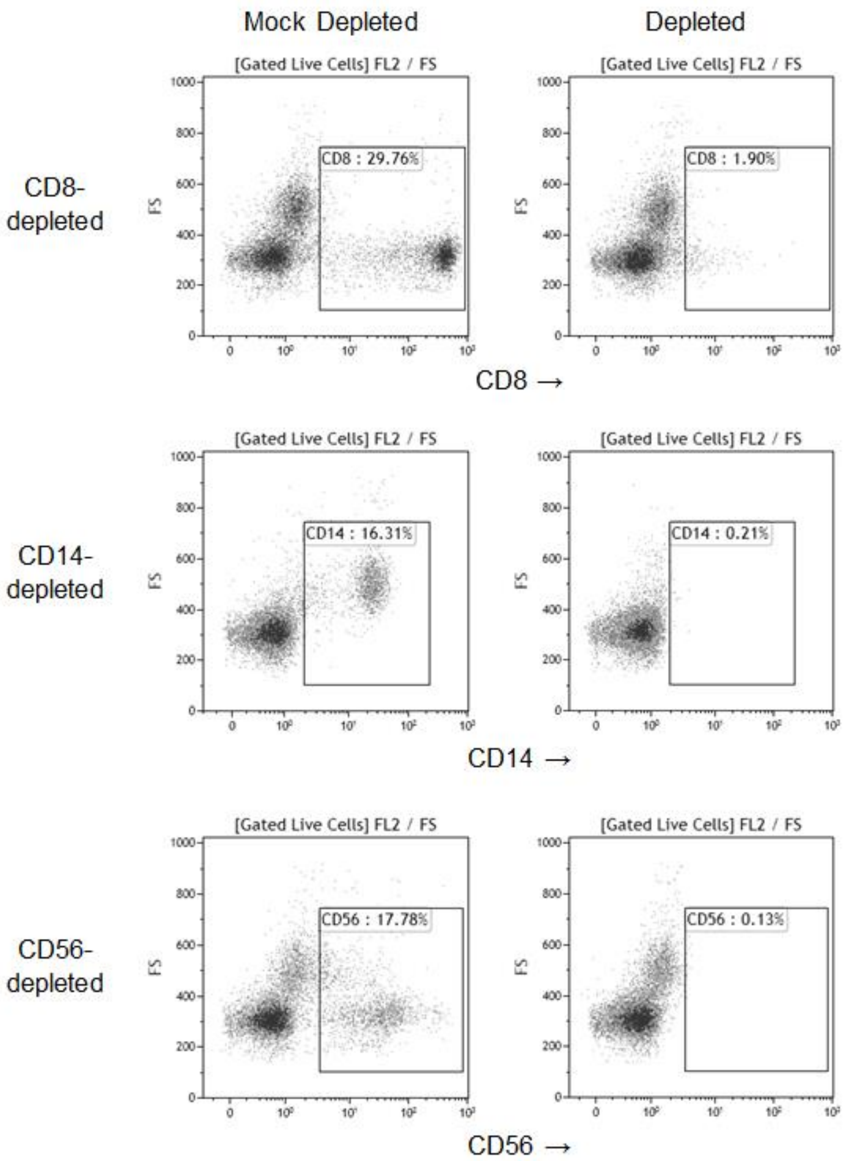

Supplemental Figure 8: Efficiency of effector subset depletion from PBMC for *in vivo* experiments. Prior to engraftment in SCID mice, PBMC were subjected to immunomagnetic bead depletion (negative selection) for: CD8 (cytotoxic T-cells), CD14 (monocytes) or CD56 (NK cells). Biotin-only conjugated beads were used for the mock-depleted condition. Efficiency of depletions was verified by flow cytometric analysis gating on viable cells (*i.e*. negative for LIVE/DEAD stain) and evaluating each of the markers. The y-axis shows forward scatter (FS).
